# An extended association screen in multiple sclerosis using 202 microsatellite markers targeting apoptosis-related genes does not reveal new predisposing factors

**DOI:** 10.1186/1477-5751-4-7

**Published:** 2005-09-05

**Authors:** René Gödde, Stefanie Brune, Peter Jagiello, Eckhart Sindern, Michael Haupts, Sebastian Schimrigk, Norbert Müller, Jörg T Epplen

**Affiliations:** 1 Department of Human Genetics, Ruhr-University, Bochum, Germany; 2 Department of Neurology, Kliniken Bergmannsheil, Ruhr-University, Bochum, Germany; 3 Department of Neurology, Knappschaftskrankenhaus, Ruhr-University, Bochum, Germany; 4 Department of Neurology, St. Josef-Hospital, Ruhr-University, Bochum, Germany; 5 Department of Transfusion Medicine, Universitätsklinikum Essen, Essen, Germany

## Abstract

Apoptosis, the programmed death of cells, plays a distinct role in the etiopathogenesis of Multiple sclerosis (MS), a common disease of the central nervous system with complex genetic background. Yet, it is not clear whether the impact of apoptosis is due to altered apoptotic behaviour caused by variations of apoptosis-related genes. Instead, apoptosis in MS may also represent a secondary response to cellular stress during acute inflammation in the central nervous system. Here, we screened 202 apoptosis-related genes for association by genotyping 202 microsatellite markers in initially 160 MS patients and 160 controls, both divided in 4 sets of pooled DNA samples, respectively. When applying Bonferroni correction, no significant differences in allele frequencies were detected between MS patients and controls. Nevertheless, we chose 7 markers for retyping in individual DNA samples, thereby eliminating 6 markers from the list of candidates. The remaining candidate, the *ERBB3 *gene microsatellite, was genotyped in additional 245 MS patients and controls. No association of the *ERBB3 *marker with the disease was detected in these additional cohorts. In consequence, we did not find further evidence for apoptosis-related genes as predisposition factors in MS.

## Introduction

Multiple sclerosis (MS) is among the most common neurological diseases of primarily of young adults [1]. It has predominantly been characterized as a chronic inflammatory disease of the central nervous system (CNS) resulting in myelin and axonal damage and the formation of focal demyelinated plaques. Myelin-reactive T cells enter the CNS via the blood-brain barrier and mediate the observed inflammatory events [2]. While the contribution of dysfunctional elements from the immune system in MS disease development has been widely accepted from the early days of MS research [3-5], the influence of neuronal death and apoptosis in acute inflammatory plaques has partially been disregarded. Yet, recent insights into the pathogenesis of MS suggest miscellaneous impacts for apoptosis in this neurological disorder, although the contribution to disease susceptibility remains elusive.

Predisposition to the disease depends on both genetic and environmental factors, as demonstrated by twin studies [6] and by virtue of the latitude-dependent geographical distribution [7], thus assigning MS to the large family of common multifactorial diseases, at least in the northern hemisphere. Despite the influence of such predisposing factors, the underlying etiopathological mechanisms as well as most genetic factors responsible for the predisposition to MS remain largely undefined. Until now, the only consistent association has been demonstrated with the HLA-DRB1*1501-DQB1*0602 haplotype in MS patients of European descent [8-10].

Apoptosis, the self-controlled death of cells, is a physiological 'suicide programme' leading to selective elimination of specific cells, either because they become dispensable in their tissue environment or harmful through infection, malignant transformation or, in general, mutation. Regarding MS, impaired apoptosis might result in elevated numbers or extended persistence of myelin-reactive T cells in the CNS tissue, enhancing the observed inflammatory processes [11,12]. On the other hand, apoptosis of neuronal cells and their glial chaperones in acute and active MS lesions has recently been demonstrated and may account for most of the disability acquired over time [13-17]. Therefore, when ascertaining candidate genes for MS association studies, factors involved in the regulation and execution of programmed cell death should be considered supplementary to those acting in the dysregulation of the immune system.

We performed an association screen in 202 microsatellite markers in or near to putative MS candidate genes related to apoptosis and the immune system using specifically designed primers and pooled DNA in a case-control design as described previously [18]. Such an 'indirect' approach strictly relies on the presence of linkage disequilibrium (LD) between certain alleles of a microsatellite marker and the corresponding predisposing mutation in the nearby candidate gene. Association was tested by means of contigency tables comparing allele frequencies in MS patients and controls. Subsequently, in case associated markers were found, we performed microsatellite genotyping of individual DNAs, thereby excluding false positive associations resulting from artifact introduced by DNA pooling.

## Materials and methods

### Patient and control DNA samples

All individuals involved in this study gave written consent for the genetic analyses. Peripheral blood samples from > 600 healthy blood donors were provided by the department of transplantation and immunology of the University hospital Eppendorf (Hamburg, Germany) and the department of transfusion medicine of the University hospital Essen (Essen, Germany). More than 800 unrelated MS patients classified according to the Poser criteria [19] and attending the Departments of Neurology, University clinic of Bochum (Germany), were included. DNA was extracted from peripheral blood leukocytes by standard methods [20]. The quality of each individual DNA was evaluated by separation on 0.7% agarose gels.

### DNA pooling

The employment of pooled DNA samples in microsatellite genotyping introduces errors [9], unless pooling is performed absolutely accurately. Concentration of DNA from each individual was quantified in triplicate using spectrophotometric measurement and then diluted to a final 50 ng/μl. After once more verifying these concentrations twice, 40 individual DNAs were combined into a DNA pool of a final concentration of 25 ng/μl. This way, 4 DNA pools were created for MS patients and controls, respectively. Using subpools prevents quantitative errors, as each allele image profile (AIP) of the respective microsatellite is statistically compared to the other subpool of the respective group.

### Microsatellite markers

Intragenic microsatellites or, if not available, microsatellites localised in the immediate vicinity (< 50 kb) of the specific gene were included. For all genes represented by microsatellite markers, oligonucleotide sequences, distances to the specific gene, and additional information are presented in the Markers website . Only markers with equal "intra-subgroup" allele distributions with ≥ 2 alleles were included in the subsequent analyses. All significantly associated markers (*p *≥ 0.05) were subsequently genotyped individually (see below).

### Tailed primer polymerase chain reaction (PCR)

We used a universal fluorescence-labelled tailed oligonucleotide added to the 5' part of the sequence-specific primer for automatic fragment analysis. The tail (5'-CATCGCTGATTCGCACAT-3') was designed to be secondary structure prone, and its sequence was "blasted" against the NCBI human genome database [21] yielding no significant homologies. Gene-specific microsatellites were chosen applying the repeat-masker option of the Santa Cruz genome browser [22]. Primers were designed and adjusted to a melting temperature of 55°C using the Primer Express 2.0 Software (ABI). Amplification was performed using three oligonucleotides: (1) a tailed forward primer (tailed F), (2) a reverse primer and (3) a labelled primer (labelled F) corresponding to the 5'-tail sequence of tailed F. PCR conditions were as follows: 1 × PCR buffer (Qiagen), 1.5 pmol labelled F, 0.2 mM each dNTP, 3 mM MgCl_2_, 0.2 pmol tailed F, 1.5 pmol reverse primer, 0.25 U Qiagen Hot Start Taq (Qiagen) and 50 ng DNA. PCR reactions were performed with an initial activation step at 95°C for 15 min; 35 cycles of denaturation at 94°C for 1 min, annealing at 55°C for 1 min and extension at 72°C for 1 min; and a final extension at 72°C for 10 min.

### Electrophoresis and genotyping

Electrophoresis was performed on a 96-well ABI377 slab-gel system. Aliquots of 1.0 μl PCR product and 2 μl of fluorescent ladder (MegaBACE ET400-R Size Standard; Amersham) were mixed. A 1 μl sample of this mix was loaded onto a 4.5% polyacrylamide (PAA) gel containing 5.625 ml 40% (19:1) PAA, 18 g urea, 5 ml 10× TBE buffer (90 mM Tris-borate, 2 mM EDTA, pH 8.3), 25 ml bidistilled H_2_O, 30 μl 10% ammoniumpersulphate and 20 μl Tetramethylethylendiamin. Prior to polymerisation, the gel mix was filtered through a 0.2-μm membrane filter. Electrophoreses were run using ABI standard protocols. Raw data were analysed using the Genotyper software (ABI), resulting in a marker-specific AIP. AIPs consist of a series of peaks with different heights that correspond to the respective allele frequency distribution within each analysed DNA pool.

### Statistics for comparison of allele frequencies

Association was tested by comparison of the MS and control AIPs. Peak heights were normalized according to the number of expected alleles per pool (n = 80). Averages of each peak (each distinct allele) were calculated according to the total allele count. Alleles with frequencies < 5% were added up and considered as one allele. Case and control distributions for combined MS and control pools, respectively, were subsequently compared statistically by means of contingency tables. Hence, *p *values are nominal and approximate because of the use of estimated rather than observed counts for allele frequencies. In order to select markers for further investigations, non-corrected *p *values were ranked according to their evidence for association [23]. Markers showing the most significant differences between MS patients and controls were subsequently chosen for further analysis by individual genotyping.

### Individual genotyping

PCR of pooled DNA samples can introduce artifacts that may cause an increased rate of false-positive results, i.e. differences between pools may appear exaggerated. Therefore, the most conspiciously differing markers were genotyped in individual DNA samples of patients and controls, both from the original pools (both n = 160) as well as additional patient and control cohorts (both n = 245) and under similar conditions as used for pool PCRs. Association was analysed by comparison of microsatellite allele frequencies from the MS cohort with the corresponding allele of the control group by chi-square testing.

## Results and discussion

The statistical evaluation of 202 microsatellite markers in 160 MS patients and 160 controls combined in 8 DNA pools, each consisting of 40 individuals, respectively, revealed 7 markers with significant differences between allele frequencies of MS patients and controls (Tab. 1).

**Table 1:** Microsatellites with significant differences (p < 0.05) in allele frequencies between MS patients and controls when screened using pooled DNA samples. No correction for multiple testing was applied here.

Microsatellite	*p *value
*NOS1*	0.0068
*NFκB2*	0.0207
*FADD*	0.0213
*GZMB*	0.0245
*ERBB3*	0.0249
*NGFβ*	0.0292
*ADPRT*	0.0335

**Figure 1:**
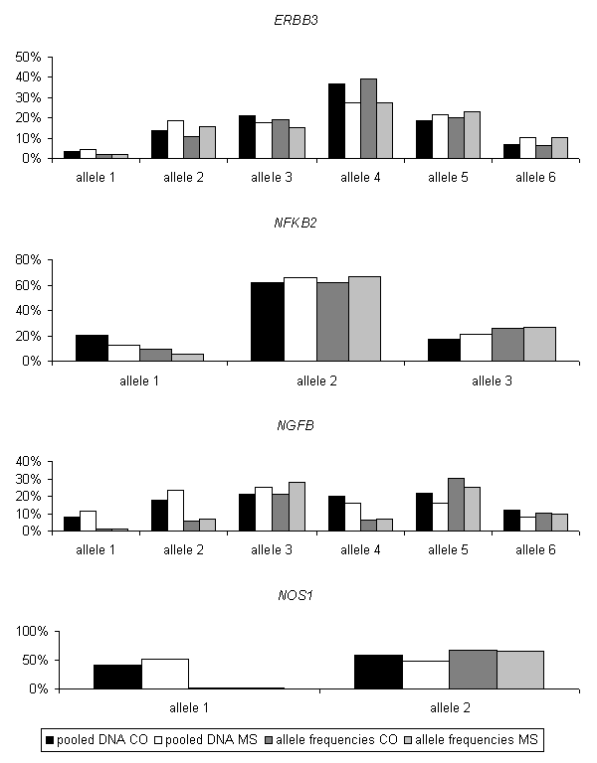
Allele frequencies genotyped in 4 microsatellite markers using pooled (black and white columns) and individual DNA samples (dark grey and light grey). CO: controls; MS: MS patients.

However, except for *NOS1*, no marker exceeded borderline significance, and Bonferroni correction for multiple testing (n = 202) did eliminate all significant results. Nevertheless, the 4 most promising markers were chosen for further analysis by individual genotyping, thereby excluding possible artifacts introduced via DNA pooling and circumventing the need for massive correction: *ERBB3 *(V-erb-b2 erythroblastic leukemia viral oncogene homologue 3), *NFκB2 *(nuclear factor of κ light polypeptide gene enhancer in B-cells 2), *NGFβ *(nerve growth factor β) and *NOS1 *(nitric oxide synthase 1). The observed allele frequencies of pooled and individual DNA samples are compared in Fig. 1.

Allele frequencies were counted from individual genotypes and compared statistically according to AIP analysis resulting from pooled DNA using the same 160 MS patients and controls. In case additional alleles were detectable, only those alleles that were observed in both experiments were analysed. The results of the statistical tests are shown in table 2.

**Table 2:** Relation of *p *values between analyses based on pooled and individual DNAs using an identical set of 160 MS patient and controls, respectively.

Microsatellite	*p *value (pools)	*p *value (individual DNAs)
*ERBB3*	0.0249	0.016*
*NFκB2*	0.0207	0.15
*NGFβ*	0.0292	0.44
*NOS1*	0.0068	0.97

*corrected for multiple testing according Bonferroni (n = 4 simultaneous tests)

Apparently, 3 of the 4 comparisons of pooled and individual DNAs show substantial differences. Only the microsatellite near to the *ERBB3 *gene remained significantly associated when the same DNA samples were retyped individually. For the *NFκB2*, *NGFβ *and *NOS1 *genes, the comparisons of allele frequencies from pooled and individually-typed DNA samples (Fig. 1) show an important and typical artifact [9]. DNA polymerases tend to preferentially amplify short alleles in favour of longer alleles (length-dependent amplification). Therefore, in PCRs based on pooled DNA samples, the shorter alleles of a microsatellite marker will often be over-represented in the resulting PCR product. This effect is most apparent in the marker *NOS1*, where one of the observed alleles in the pooled experiment obviously results exclusively from the abovementioned effect. Also in *NGFβ*, the alleles 1 and 2 were significantly over-represented in the screen using pooled DNA, resulting in a false positive association. Only for the *ERBB3 *gene, the observed allele frequencies in the typing experiment based on pooled DNA adequately correspond to the individually typed frequencies.

**Figure 2:**
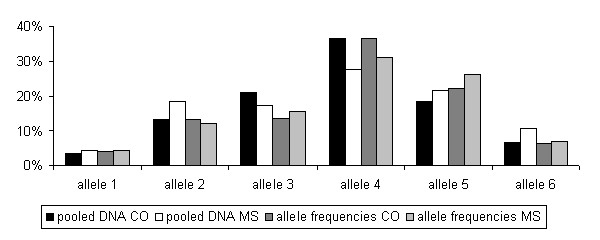
Allele frequencies genotyped in the microsatellite ERBB3 using the originally pooled (black and white columns) and the additional 490 individual DNA samples (dark grey and light grey). CO: controls; MS: MS patients.

In order to validate the association of *ERBB3 *with MS, we performed genotyping of another cohort of 245 MS patients and controls, respectively (frequencies shown in Fig. 2).

Statistical analysis of the latter allele frequency distribution revealed a non significant *p *value of 0.325. Therefore, the association of the *ERBB3 *microsatellite could not be confirmed in the additional DNA cohorts of MS patients and controls.

In conclusion, we did not find supporting evidence for involvement of apoptosis-related genes in the predisposition to MS. Nevertheless, such a contribution cannot be excluded based exclusively on our experiments for various reasons. Only a fraction of all apoptosis-related genes has been included in our survey and, therefore, many more genes may represent auspicious candidates. Moreover, as our approach depends solely on the presence of LD between a marker and a predisposing mutation, missing LD between the microsatellite and its corresponding gene will also cause a negative result. As the HapMap-Project [24] progresses rapidly and, therefore, information about the haplotype block structure of the human genome increases substantially, it might soon be possible to reappraise our negative results with respect to the haplotype block structure of the gene under examination.
